# **PANCREATODUODENECTOMY: BRAZILIAN PRACTICE PATTERNS** *

**DOI:** 10.1590/0102-6720201700030007

**Published:** 2017

**Authors:** Orlando Jorge M TORRES, Eduardo de Souza M FERNANDES, Rodrigo Rodrigues VASQUES, Fabio Luís WAECHTER, Paulo Cezar G. AMARAL, Marcelo Bruno de REZENDE, Roland Montenegro COSTA, André Luís MONTAGNINI

**Affiliations:** 1 Department of Surgery, Federal University of Maranhão, São Luis, MA; 2 Federal University of Rio de Janeiro, Rio de Janeiro, RJ; 3 Santa Casa Hospital, Porto Alegre, RS; 4 São Rafael Hospital, Salvador, BA; 5 Santa Marcelina Hospital, São Paulo,SP; 6 Santa Lucia Hospital, Brasilia, DF; 7 University of São Paulo Medical School, São Paulo, SP, Brazil.

**Keywords:** Pancreatoduodenectomy, Pancreaticoduodenectomy, Whipple, Technique

## Abstract

**Background:**

Pancreatoduodenectomy is a technically challenging surgical procedure with an incidence of postoperative complications ranging from 30% to 61%. The procedure requires a high level of experience, and to minimize surgery-related complications and mortality, a high-quality standard surgery is imperative.

**Aim:**

To understand the Brazilian practice patterns for pancreatoduodenectomy.

**Method:**

A questionnaire was designed to obtain an overview of the surgical practice in pancreatic cancer, specific training, and experience in pancreatoduodenectomy. The survey was sent to members who declared an interest in pancreatic surgery.

**Results:**

A total of 60 questionnaires were sent, and 52 have returned (86.7%). The Southeast had the most survey respondents, with 25 surgeons (48.0%). Only two surgeons (3.9%) performed more than 50% of their pancreatoduodenectomies by laparoscopy. A classic Whipple procedure was performed by 24 surgeons (46.2%) and a standard International Study Group on Pancreatic Surgery lymphadenectomy by 43 surgeons (82.7%). For reconstruction, pancreaticojejunostomy was performed by 49 surgeons (94.2%), single limb technique by 41(78.9%), duct-to-mucosa anastomosis by 38 (73.1%), internal trans-anastomotic stenting by 26 (50.0%), antecolic route of gastric reconstruction by 39 (75.0%), and Braun enteroenterostomy was performed by only six surgeons (11.5%). Prophylactic abdominal drainage was performed by all surgeons, and somatostatin analogues were utilized by six surgeons (11.5%). Early postoperative enteral nutrition was routine for 22 surgeons (42.3%), and 34 surgeons (65.4%) reported routine use of a nasogastric suction tube.

**Conclusion:**

Heterogeneity was observed in the pancreatoduodenectomy practice patterns of surgeons in Brazil, some of them in contrast with established evidence in the literature.

## RESUMO

**Racional:**

A duodenopancreatectomia é um procedimento cirúrgico tecnicamente desafiador, com uma incidência de complicações pós-operatórias variando de 30% a 61%. O procedimento requer experiência de alto nível, e para minimizar complicações relacionadas à cirurgia uma padronização de alta qualidade é imperativa.

**Objetivo:**

Compreender o padrão da prática brasileira para duodenopancreatectomia.

**Método:**

Um questionário foi elaborado com a finalidade de obter uma visão geral da prática cirúrgica em câncer do pâncreas, treinamento específico e experiência em duodenopancreatectomia. O questionário foi enviado para cirurgiões com declarado interesse em cirurgia pancreática.

**Resultados:**

Um total de 60 questionários foi enviado e 52 retornaram (86,7%). A região sudeste foi a que mais respondeu, com 25 cirurgiões (48,0%). Apenas dois cirurgiões (3,9%), realizaram mais do que 50% das duodenopancreatectomia por videolaparoscopia. O procedimento clássico de Whipple foi realizado por 24 cirurgiões (46,2%) e a linfadenectomia padrão do Grupo Internacional de Estudo em Cirurgia Pancreática foi realizada por 43 cirurgiões (82,7%). Para a reconstrução, a pancreatojejunostomia foi realizada por 49 cirurgiões (94,2%), em alça única por 41 (78,9%), com anastomose do tipo ducto-mucosa por 38 (73,1%). O cateter transanastomose foi realizado por 26 cirurgiões (50%), reconstrução gástrica antecólica por 39 (75%) e enteroanastomose tipo Braun apenas por seis cirurgiões (11,5%). A drenagem abdominal profilática foi realizada por todos os cirurgiões e o uso de análogos da somatostatina por seis cirurgiões (11,5%). Nutrição enteral precoce no pós-operatório foi utilizada de rotina por 22 cirurgiões (42,3%) e 34 cirurgiões (65,4%), usaram sonda nasogástrica de rotina.

**Conclusão:**

Heterogeneidade foi observada na prática padrão da duodenopancreatectomia pelos cirurgiões no Brasil e, algumas delas em contraste com evidências estabelecidas na literatura.

## INTRODUCTION

Ductal adenocarcinoma of the pancreatic head is the fourth leading cause of cancer-related deaths worldwide, and surgical resection by pancreatoduodenectomy (PD) is the only potential cure. PD is a technically challenging surgical procedure, and the postoperative mortality is currently 3-5% in experienced centers. However, the incidence of postoperative complications remains high, ranging from 30-61%. The most common complications include delayed gastric emptying, postoperative pancreatic fistula, postoperative bleeding, and infectious complications. Surgical technical factors in the resection and reconstruction during PD have been implicated in the development of these complications^7^ ^,^ ^9^ ^,^ ^39^. The procedure requires a high level of experience and standards with regard to technical aspects as well as perioperative care. Minimization of surgery-related mortality and morbidity is imperative, and an important feature of high-quality standard surgery^9^ ^,^ ^28^ ^,^ ^38^. Centralization of highly complex surgery has been discussed extensively during the past decade and in pancreatic resection has been associated with a decrease in hospital mortality and costs. The differences in outcome can be explained by many variables, and surgeon-volume and hospital-volume are important factors in morbidity and mortality rates^11^ ^,^ ^38^. 

There is significant heterogeneity in the practice patterns of PD worldwide. The best ways to resect the tumor and restore pancreatic digestive continuity remain controversial^9^ ^,1128,^ ^38^. Technical advancements in the surgical management of pancreatic cancer have improved perioperative morbidity and mortality. The International Study Group on Pancreatic Surgery (ISGPS) established standardized definitions for postoperative pancreatic fistula, delayed gastric emptying (DGE), and standard lymphadenectomy. These definitions have an important role in surgical decisions and comparisons^3^ ^,^ ^37^ ^,^ ^43^_._

The aim of this study was to analyze the Brazilian practice patterns for PD. 

## METHOD

This study was supported by the Brazilian Chapter of the International Hepato-Pancreato Biliary Association (CB-IHPBA), and permission was obtained from the president of the CB-IHPBA in order to contact the membership and send the questionnaires. The survey was sent to members who declared an interest in pancreatic surgery. A total of 60 questionnaires were sent and after four weeks, 52 were returned, yielding a response rate of 86.7%. 

The questionnaire was designed to obtain an overview of the clinical practice in pancreatic cancer, and the components included region of practice, specific training, and experience in PD. All responses were collected anonymously, and the surgeons indicated their region of practice as North, Northeast, Center-West, Southeast, and South. The specific training and experience included the specialty, the hospital as academic or private, years of experience in PD, total number of PDs, and the number of PDs performed last year. The participants were asked about technical aspects including incision, laparoscopy, resection, reconstruction, the use of stenting, and abdominal drainage. Clinical aspects included the use of somatostatin, use of a nasogastric suction tube, and nutritional support. 

## RESULTS

A total of 60 surgeons from Brazil were invited to participate, and 52 (86.7%) returned the questionnaire. The respondents according to geographic region were North 2 (3.9%), Northeast 10 (19.2%), Center-West 4 (7.7%), Southeast 25 (48.0%), and South 11 (21.2%). All surgeons actively performed PD, and most surgeons described themselves as hepato-pancreatobiliary surgeons (30-57.7%) with their activities in both public and private hospitals (36-69.2%). Regarding the experience in PD, 34.6% of respondents had performed the procedure for over 20 years. Seventy-three percent of participants had performed more than 50 PDs, and 28.8% had performed more than 20 PDs during the previous year (Table 1). 

**TABLE 1:** Characteristics of study population (n and %)

Specialty/Training	Experience	Number of PDs	DPs em 2015	Practice setting
General Surgery 4 (7.7)	Practice (PD) in years	1-20 2 (3.9)	1-5 6 (11,5)	Public (academic/university) 3 (5.8)
GI Surgery 8 (15.4)	0-5 0 (0)	21-50 12 (23.1)	6-10 14 (27,0)	Public(non-academic/non-university) 3 (5.8)
Surgical Oncology 9 (17.3)	6-10 9 (17.3)	51-100 16 (30.8)	11-15 9 (17,3)	Public and private 36 (69.2)
Hepato-pancreatobiliary 30 (57.7)	11-15 13 (25.0)	101-150 6 (11.5)	16-20 8 (15,4)	Private only 10 (19.2)
Pancreatic Surgery 1 (1.9)	16-20 12 (23.1)	151-200 2 (3.9)	21-25 6 (11,5)	
	>20 18 (34.6)	201-300 7 (13.4)	26-30 3 (5,8)	
		> 300 7 (13.4)	> 30 6 (11,5)	

**FIGURE 1:**
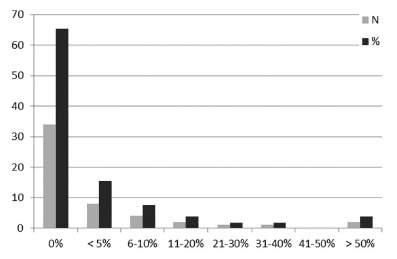
Laparoscopic pancreatoduodenectomy (%)

Most of the surgeons (65.4%) performed only open conventional surgery, and only two surgeons (3.9%) performed more than 50% of their PDs by laparoscopy (Figure 1). The incisions performed were bilateral subcostal rooftop incision (57.7%), midline incision (17.3%), modified Makuuchi incision (9.6%), and others (15.4%). 

**FIGURE 2:**
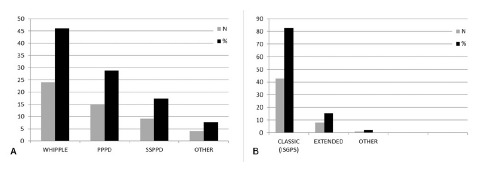
Resection (A) and Lymphadenectomy (B)

Heterogeneity was observed regarding the pancreatic resection. A classic Whipple procedure was performed by 24 surgeons (46.2%), pylorus-preserving PD (PPPD) by 15 (28.8%), and subtotal stomach-preserving PD (SSPPD) by nine surgeons (17.3%). Most surgeons (43-82.7%) performed standard ISGPS lymphadenectomy (Figure 2A and 2B). 

**FIGURE 3:**
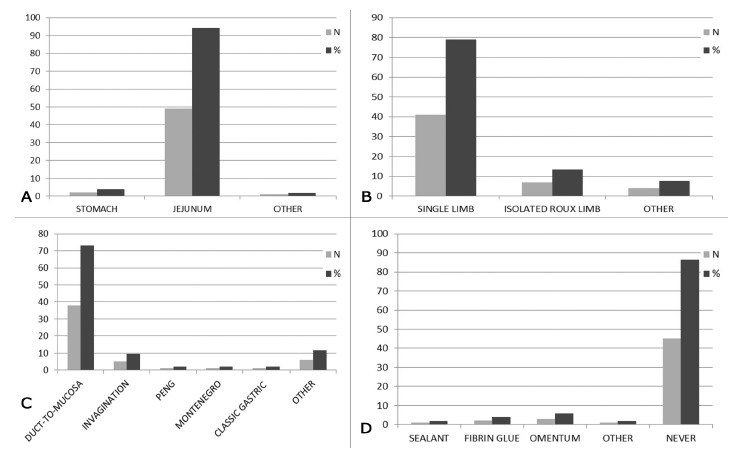
Pancreatic anastomosis and protection (A-D)

 For reconstruction, a large proportion of respondents (49-94.2%) reported that they always perform pancreatojejunostomy rather than pancreatogastrostomy; 49 (78.9%) surgeons performed a single limb anastomosis. There was a great variability regarding pancreatic anastomosis. Thirty-eight surgeons (73.1%) reported using the duct-to-mucosa method, five performed invagination; nine performed other techniques including the Peng and Montenegro techniques for pancreatogastrostomy or pancreatojejunostomy. Internal drainage of the pancreatic duct with a trans-anastomotic stent was performed by 26 surgeons (50.0%), external stent was performed by two surgeons (3.9%) and 24 surgeons (46.1%) never use stent. The majority of surgeons (45-86.5%) did not employ any maneuver to protect the anastomosis in order to reduce the rates of pancreatic fistula (Figure 3A-D).

**FIGURE 4:**
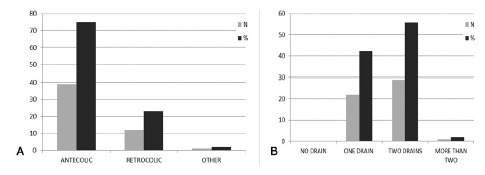
Gastric reconstruction (A) and abdominal drainage (B)

The antecolic route of gastric reconstruction was performed by a large proportion of respondents (39-75.0%); Braun enteroenterostomy was an uncommon procedure among the Brazilian surgeons, performed by only six respondents (11.5%). Prophylactic abdominal drainage was routine for all surgeons, and the majority (29-55.8%) used two drains (Figure 4A and 4B). 

**FIGURE 5:**
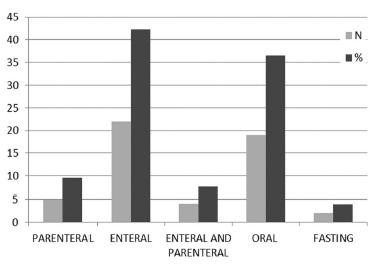
Early feeding

Interestingly, only a small number of participants use somatostatin analogues to prevent postoperative pancreatic fistula (6 surgeons - 11.5%). Early postoperative enteral nutrition was routinely employed by 22 surgeons (42.3%), but 36.5% (19 surgeons) preferred early oral intake (Figure 5). Thirty-four surgeons (65.4%) reported routinely using a nasogastric suction tube for decompression. 

## DISCUSSION

Significant heterogeneity was observed in the PD practice patterns of surgeons in Brazil. Most responders (48%) were from the Southeast, which is the most developed region of the country. One surgeon (1.9%) described himself as a pancreatic surgeon, but the majority described themselves as hepato-pancreatobiliary surgeons. 

According to some studies, a high-volume surgeon must perform ≥20 PDs per year, resulting in a greater number of resected lymph nodes and reduced intraoperative blood loss and operative time, and the learning curve for PD surgeons with a career volume of ≥50 cases. Thereafter, the surgeon achieves a better postoperative outcome, including reduced operative time and duration of hospital stay^11^ ^,^ ^28^ ^,^ ^38^.

In the current study, 38 surgeons (73.0%) performed ≥50 PDs, but only 12 surgeons (28.8%) performed >20 PDs last year, defined as high volume. Twenty-six surgeons defined as experienced surgeons (>50 PDs) performed <20 procedures in 2015. PD is a highly complex procedure, and previous studies have demonstrated that increased volume of this procedure leads to a decrease in perioperative morbidity and mortality^11^ ^,^ ^28^ ^,^ ^38^. Centralization of pancreatic surgery should be discussed in Brazil in order to define a pattern of care and improve outcomes.

In the current study, 34 surgeons (65.4%) had experience with laparoscopic PD, and only two surgeons (3.9%) performed more than half of these procedures by laparoscopy. Laparoscopic PD is one of the most sophisticated operations in gastrointestinal surgery. In a systematic review and meta-analysis of minimally invasive versus open pancreaticoduodenectomy, Zhang et al.^45^ and Rooij et al.^34^ concluded that minimally invasive surgery is associated with a reduction in estimated blood loss, delayed gastric emptying, and a shorter hospital stay. However, the operative time is much longer than that of an open approach, higher mortality was observed in low-volume centers, and this procedure should be implemented only in centers with a structured training program^34^ ^,^ ^45^. 

Laparoscopic PD has not been included as a standard therapeutic approach for pancreatic surgeons. Its acceptance is still slow owing to inherent technical limitations and the need for skills in advanced laparoscopic surgery. However, this procedure is safe and feasible. The selection of the patients, experience in open PD, and skill in advanced laparoscopic surgery are important factors for the success of this procedure^8^ ^,^ ^40^. 

An important issue analyzed in the present study is related to the decision to preserve the pylorus during PD. Three main forms of PD have been described. The first and classic PD was developed by Whipple and included distal hemigastrectomy; the second is pylorus-preserving pancreatoduodenectomy (PPPD) described for benign disease; and recently, the third is subtotal stomach-preserving pancreatoduodenectomy (SSPPD). Some complications are related to the classic Whipple procedure, such as dumping syndrome and diarrhea, and others complications are related to PPPD, such as DGE in 19% to 61%, inadequate lymphadenectomy, and long term nutritional deficiency^7^ ^,^ ^9^ ^,^ ^39^. In a meta-analysis by Iqbal et al.^19^, the authors observed no differences in complications between the classic Whipple procedure and PPPD. 

In a prospective, randomized, controlled trial, Kawai et al.^21^ observed that the incidence of delayed gastric emptying was lower in the SSPPD group than in the PPPD group (p=0.024). Fujii et al.^15^ observed that preservation of the pyloric ring has little value in surgery for pancreatic head cancer and when regional lymphadenectomy is necessary, SSPPD instead of PPPD might be recommended in order to reduce the incidence of postoperative DGE. In the current study, we observed that the classic Whipple procedure was performed by 46.2% (24 surgeons), pylorus preserving by 28.8% (15), and SSPPD by 17.3% (nine). SSPPD is evolving worldwide, preserving the stomach and supporting the evidence that the pylorus is related to complications such as delayed gastric emptying, postoperative malnutrition at one year, and incomplete lymphadenectomy in up to 9.0% of patients^15^ ^,^ ^21^ ^,^ ^41^. Recently introduced in Brazil, SSPPD is gradually replacing the two previous procedures. 

Lymph node metastases adversely affect survival in patients with pancreatic adenocarcinoma, and lymphadenectomy plays an important role to identify patients who might benefit from adjuvant treatment. The ISGPS described the extent and nodal stations that should be removed in standard lymphadenectomy for PD. The lymph nodes stations include 5, 6, 8a, 12b1, 12b2, 12c, 13a, 13b, 14a, 14b, 17a, and 17b. Furthermore, the number of lymph nodes retrieved in the standard PD should be ≥15 for adequate pathologic staging of the disease and the lymph node ratio ≤0.2^37^. 

Extended lymphadenectomy has no impact on long-term oncological outcomes after PD. Extended lymphadenectomy is associated with increases in operative time, increased requirements for blood transfusions, and a greater incidence of overall complications. Morbidity related to extended lymphadenectomy includes diarrhea, delayed gastric emptying, and malnutrition due to denervation of the celiac plexus and the plexus around the superior mesenteric artery. Standard lymphadenectomy is associated with reduced morbidity and the same long-term benefits^10^ ^,^ ^32^.

In the present study, eight surgeons (15.4%) still performed extended lymphadenectomy. According to the ISGPS, there is no evidence of benefit from extended lymphadenectomy. Therefore, this procedure cannot be recommended and should not be applied for patients with pancreatic head adenocarcinoma requiring PD^37^. 

An important operative issue is the reconstruction of the pancreatic stump, and pancreatic fistula is the most important postoperative complication after PD. According to the ISGPS, postoperative pancreatic fistula is defined as amylase levels in the effluent three times greater than the plasma levels after postoperative day three^3^. To reduce the incidence of pancreatic fistula, some variations of pancreaticojejunostomy have been described. The two more common methods of pancreaticoenteric anastomosis are conventional pancreaticojejunostomy, in which the same jejunal limb is used for the pancreatic, biliary, and gastric anastomosis, and isolated Roux limb pancreaticojejunostomy^22^ ^,^ ^24^_._ Machado et al.^24^ reported the concept of isolated Roux limb pancreatojejunostomy in 1976 in an attempt to reduce anastomotic fistula and avoid mortality if a fistula occurred. In their series, two patients developed pancreatic fistula without mortality^24^. 

The argument in favor of using a Roux limb is the diversion of bile away from the pancreatojejunostomy to minimize activation of pancreatic enzyme and reduce the risk of fistula formation; if this complication occurs, it will be a pure pancreatic fistula with less repercussion. Some disadvantages of an isolated Roux limb are an additional anastomosis and increased operative time^10^ ^,^ ^24^.

Compared to single limb pancreatojejunostomy after PD, isolated Roux limb pancreatojejunostomy does not offer any advantages in terms of pancreatic fistula, duration of spontaneous closure, and associated mortality, and the Roux limb makes the entire procedure longer and more complex^10^ ^,^ ^24^.

Many factors are responsible for pancreatic fistula, including disease factors, patient-related factors, surgeon-related factors, and operative factors. Regarding operative factors, the type of pancreatic anastomosis is chosen according to a surgeon’s experience^3^. For reconstruction of the pancreas remnant after PD, two main methods of anastomosis have been described: pancreatogastrostomy and pancreatojejunostomy^22^ ^,^ ^29^.

In a previous meta-analysis of seven randomized controlled trails, Liu et al.^23^ demonstrated that pancreatogastrostomy is more efficient than pancreatojejunostomy in reducing the incidence of clinically relevant pancreatic fistula. However, the morbidity and mortality were comparable^23^. Others recent studies confirm the superiority of pancreatogastrostomy over pancreatojejunostomy in reducing pancreatic fistula and decreasing its severity^22^ ^,^ ^23^ ^,^ ^29^. Some technical advantages of pancreatogastrostomy are the excellent blood supply, the thickness of the stomach wall, avoidance of a long jejunal loop, and protection of the anastomosis from auto-digestion by the acidic gastric environment by pancreatic enzyme inactivation. The Montenegro technique was first described by a Brazilian surgeon in 2005 and presented at the European Hepato-Pancreato-Biliary Association Congress, Heidelberg (Germany)^23^. 

The position statement of the ISGPS on pancreatic anastomosis after PD concluded that neither the use of pancreaticogastrostomy nor pancreaticojejunostomy has been shown to result in a substantial difference in the incidence of postoperative pancreatic fistula after a pancreatoenteric anastomosis^31^.

Pancreatic anastomosis is difficult to perform in the soft pancreas and a small duct, even by experienced surgeons^2^ ^,^ ^5^ ^,^ ^6^. In the current study, duct-to-mucosa anastomosis was performed by 38 surgeons (73.1%) and invagination by five surgeons (9.6%). Some studies have shown that the duct-to-mucosa pancreatojejunostomy in patients with a pancreatic duct diameter more than 3 mm or a hard pancreas is associated with a lower rate of pancreatic fistula. However, in patients with a soft pancreas, this advantage was not observed^5^ ^,^ ^6^.

Pancreatic reconstruction should be performed according to the pancreatic texture and diameter of the pancreatic duct in order to overcome complications related to the type of anastomosis^6^. In a prospective, randomized, dual-institution trial, Berger et al.^5^ observed that the rate of pancreatic fistula was higher for patients undergoing duct-to-mucosa reconstruction^5^. A prospective randomized trial by Bassi et al.^2^ evaluated a duct-to-mucosa versus an end-to-side pancreatojejunostomy. They observed no differences in complications between the groups^2^.

According to El Nakeeb et al.^13^, the rate of pancreatic fistula was not significantly different between the duct-to-mucosa pancreatojejunostomy group and the invagination pancreatojejunostomy group. However, the median operative time for reconstruction was significantly longer with duct-to-mucosa pancreatojejunostomy. Moreover, postoperative steatorrhea was more common after duct-to-mucosa pancreatojejunostomy^13^. Two authors in the current study (OJMT and RRV) performed pancreatic anastomoses according to the Shrikhande technique^35^. 

During the early period after PD, gastrointestinal peristaltic function is not completely restored, and retention of bile and pancreatic juice in the jejunum can occur. These digestive fluids can promote tension on the anastomosis and increase the risk of pancreatic fistula. Placement of stents into the pancreatic duct is intended to improve the outflow of pancreatic juice during the early period after pancreatojejunostomy and reduce pancreatic fistula. The other indication is for a more precise placement of sutures during pancreatic anastomosis^28^ ^,^ ^30^.

In the current study, 26 surgeons (50%) preferred internal stenting during pancreatic anastomosis, two surgeons (3.9%) performed external stenting, and 24 surgeons (46.1%) never use a stent. The use of a stent in the pancreatic duct can provide internal or external (out of the body) drainage. Internal drainage dos not result in loss in pancreatic enzymes, and the drainage tube should be 10-20 cm long and cross the biliary intestinal anastomosis^28^ ^,^ ^30^. Pessaux et al.^33^ observed that external drainage can significantly reduce the incidence of pancreatic fistula in patients with a relatively soft pancreas and without duct dilatation (<3 mm in diameter). Moreover, when pancreatic fistula occurs, the volume is low and the severity of complications is reduced. The external drain must be removed after 2-3 weeks^33^. In conclusion, external stenting has the advantage of reducing the incidence of pancreatic fistula compared with no stenting and internal stenting. 

Delayed gastric empting was defined in 2007 by the ISGPS, and its incidence is 21% according to Cameron et al.^7^, local ischemia, nerve damage, and spasm of the pylorus after pylorus-preserving PD are important causes, and the incidence of DGE is now improving after SSPPD. Torsion or angulation of the gastroenteric reconstruction has been described as another important technical aspect^7^ ^,^ ^21^ ^,^ ^43^. 

Two routes are usually used for gastric reconstruction after PD: the antecolic route and the retrocolic route. Several studies support an association between the reconstruction route and the incidence of DGE. From a mechanical point of view, with antecolic reconstruction, torsion or angulation of the anastomosis can be avoided because the distal stomach and descending jejunal limb are aligned^4^ ^,^ ^20^. In a recent meta-analysis, Bell et al.^4^ observed that the antecolic rout is associated with a lower incidence of DGE, reduced length of stay, and early resumption to oral intake^4^. 

In a prospective randomized clinical trial, Imamura et al.^18^ observed that the incidence of DGE was not significantly different between the procedures. However, they concluded that vertical retrocolic duodenojejunostomy is an acceptable procedure for a lower incidence of DGE^18^. Others systematic reviews and meta-analyses concluded that the antecolic reconstruction route might be associated with a reduction in postoperative hospital stay and early oral intake, but it might not decrease the incidence of DGE^4^ ^,^ ^18^ ^,^ ^20^. Therefore, the route of reconstruction after PD should be selected according to the surgeon’s preference. 

Braun enteroenterostomy is an anastomosis between the afferent and efferent limbs. It is designed to divert bile and pancreatic juice from the afferent limb in order to reduce reflux into the stomach. In theory, after PD, Braun enteroenterostomy potentially stabilizes the anastomosis, helps prevent twisting and angulation, and diverts biliary and pancreatic juices away from the stomach^44^ ^,^ ^46^. A systematic review and meta-analysis of retrospective studies by Xu et al.^44^, concluded that Braun enteroenterostomy is associated with a decreased incidence of DGE, nasogastric tube reinsertion, and postoperative emesis^44^. A retrospective study by Zhang et al.^46^ found no significant difference between patients with or without Braun enteroenterostomy^46^. In conclusion, a prospective, randomized trial is necessary to evaluate the benefits of Braun enteroenterostomy.

Some techniques have been employed to protect the anastomosis and reduce complications like pancreatic fistula. The use of fibrin glue, omentum, and sponge has been described^28^. In the current study, 45 surgeons (86.5%) never used anastomotic protection. Some studies including that of the ISGPS found no benefit in reducing the rate of pancreatic fistula. Furthermore, the costs of these sealants and glue are high^25^ ^,^ ^36^. Therefore, commercialized products to protect the anastomosis are not recommended. 

In the present study, 46 surgeons (88.5%) did not use octreotide routinely. In a brilliant study performed by McMillan et al.^26^, prophylactic octreotide was associated with a greater incidence of clinically relevant pancreatic fistula, re-operation, and increased length of hospital stay. The explanations by the authors are that octreotide can decrease pancreatic perfusion and gastroduodenal blood flow, impair wound healing at the site of the anastomosis, and suppress the secretion of anabolic and tropic hormones that improve the wound-healing process^26^. However, in a prospective randomized, double-blind trial, Allen et al.^1^ found that perioperative treatment with pasireotide decreased the rate of clinically significant postoperative pancreatic fistula^1^. The ISGPS concluded that routine use of octreotide might be relevant only in high-risk patients^36^.

Prophylactic abdominal drainage at the operative bed after PD is based on the rationale that it contributes to the elimination of abdominal fluid and to the early detection of complications in the abdominal cavity. However, some surgeons have proposed that prophylactic abdominal drainage should not be routine practice after PD^12^ ^,^ ^27^ ^,^ ^28^ ^,^ ^42^. In the current study, abdominal drainage was utilized by all the surgeons. 

A systematic review and meta-analysis by Dou et al.^12^ observed that prophylactic abdominal drainage does not improve morbidity, pancreatic fistula, and length of hospital stay. However, the elimination of prophylactic abdominal drainage is unsafe and results in an increase in the mortality rate^12^. In a randomized, prospective, multicenter trial, Van Buren II et al.^42^ concluded that the elimination of intraperitoneal drainage increases the frequency and severity of complications if adopted in all cases of PD^42^. Callery et al.^6^ proposed a clinical risk score to predict postoperative pancreatic fistula after PD, and in a randomized, prospective, multi-institutional study, McMillan et al.^27^ concluded that abdominal drainage could be avoided in the approximately one-third of patients with a negligible or low risk for pancreatic fistula^27^. The ISGPS suggests that prophylactic abdominal drainage should be avoided in patients with a negligible/low risk for postoperative pancreatic fistula^36^.

After pancreatic surgery, placement of a nasogastric tube for decompression has been a standard procedure. This procedure is thought to be necessary to prevent vomiting and gastric distension, reduce the risk of anastomotic fistula, and increase patient comfort. The high incidence of delayed gastric emptying after pancreaticoduodenectomy has stimulated the use of a nasogastric tube^7^ ^,^ ^19^ ^,^ ^39^. In the current study, 65.4% (34 surgeons) used a postoperative nasogastric tube. 

In other areas of gastrointestinal surgery, the use of a nasogastric tube has been proven unnecessary. The presence of a nasogastric tube is associated with an increased risk of post-operative pulmonary complications and does not decrease the incidence of any other complications. Some studies were not able to demonstrate advantages in the outcome after PD with the routine use of a postoperative nasogastric tube. In patients without a nasogastric tube, the return of bowel function is faster, the interval to first oral intake is less, and lack of a nasogastric tube is not associated with an increase in anastomotic fistula^9^ ^,^ ^14^ ^,^ ^28^.

Some complications, such as esophagitis, pharyngitis, injuries to the larynx, and electrolytic losses have been reported. In addition, use of a nasogastric tube has been associated with anastomotic fistula, wound infection, and incisional hernia^14^ ^,^ ^28^ ^,^ ^39^. Therefore, the routine use of a nasogastric tube after PD should be reconsidered. 

PD is commonly performed in malnourished patients, and malnourishment this situation, if severe, can be associated with a higher incidence of complications. Postoperative nutritional support is important to improve the clinical outcome and nutrition status of patients^7^ ^,^ ^9^ ^,^ ^16^ ^,^ ^17^ ^,^ ^28^. In the current study, 42.3% (22 surgeons) preferred early enteral nutrition, and 36.5% (19) preferred early oral nutrition. 

Gerritsen et al.^16^ performed a retrospective study to evaluate the efficacy and complications of jejunostomy, nasojejunal tube, and total parenteral nutrition after PD. None of these strategies was found to be superior in terms of the time to start normal oral intake, morbidity, and mortality. Some studies concluded that postoperative early oral feeding is a clinically feasible, safe, and effective method of nutritional support. Moreover, compared to traditional management, early oral feeding reduces the time to resumption of adequate oral intake and length of hospital stay without increasing complication rates^16^ ^,^ ^17^.

## CONCLUSIONS

Heterogeneity was observed in the PD practice patterns of surgeons in Brazil, and some of the practice patterns were in contrast with established evidence in the literature. The hospital and surgeon volume, patterns in resection and reconstruction, experience in laparoscopic advanced surgery, and perioperative management might contribute to different outcomes. Centralization, which has been adopted in some countries and has some advantages, should be discussed in Brazil. 
